# The potential of utilizing Provitamin A‐biofortified maize in producing *mutwiwa*, a Zimbabwean traditional fermented food

**DOI:** 10.1002/fsn3.2125

**Published:** 2021-01-18

**Authors:** Armistice Chawafambira, Qhubekani Nyoni, Tafadzwa Mkungunugwa

**Affiliations:** ^1^ Department of Food Science and Technology Chinhoyi University of Technology Chinhoyi Zimbabwe; ^2^ Standards Association of Zimbabwe Harare Zimbabwe

**Keywords:** essential amino acid, malnutrition, milk, *Mutwiwa*, Provitamin A‐biofortified maize, sub‐Saharan Africa

## Abstract

Biofortification interventions have the potential to combat micronutrient deficiencies, such as vitamin A deficiency (VAD), which is prevalent in Zimbabwe. The poor acceptability of provitamin A (PVA)‐biofortified maize is still a challenge that exists in Zimbabwe. This study investigated the effect of replacing white maize (WM) with PVA‐biofortified maize on the nutritional composition of *mutwiwa,* a Zimbabwean traditional food, and its microbiological safety. Chemical and microbiological tests were conducted using AOAC standard methods. Total carotene content was 12.78 µg/g dry weight (DW) in PVA‐biofortified maize and 1.52 µg/g DW in WM. The proximate composition of PVA‐biofortified *mutwiwa* (PVABM) was 5.2, 28.6, 2.1, 62.2, and 2.0 g/100 g wet basis (w.b) for protein, carbohydrates fiber, moisture, and ash, respectively. Total soluble solids, β‐carotene, vitamin C, and vitamin A contents were 3.6 ^o^Brix, 110 µg/100 g, 0.54 mg/100 g, and 9 µg REA/100 g, respectively. Lysine, phenylalanine, and histidine contents were 0.71, 1.15, and 0.56 g/100 g w.b, respectively. Iron, calcium, phosphorus, and zinc content were 7.8, 60.5, 410.8, and 60 mg/100 g w.b, respectively. Mesophilic bacteria, lactic acid bacteria, coliforms, yeast, and molds were all <1 Log CFU/ml. The nutritional, amino acid and mineral contents were significantly different (*p* < .05). In conclusion, the results of this study were satisfying and recommend the processing of PVA‐biofortified maize as a potential strategy to combat VAD and mineral malnutrition in Zimbabwe and other regions in Sub‐Saharan Africa.

## Graphical abstract

Provitamin A‐biofortified maize can be processed into traditional foods with the potential to combat vitamin A and nutrition malnutrition in Africa.

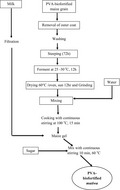

## 1 INTRODUCTION

Biofortification is a scientific process that aims to improve the nutrient content of plants by performing plant breeding or recombinant DNA technology (rDNA) (Biofortification of Staple Crops., 2018; Govender et al., 2019). Biofortified crops have great potential in improving the nutritional status of vulnerable population groups in cases where conventional fortification, supplementation, and other dietary strategies are limited (Biofortification of Staple Crops., 2018). Research and development on the uses of biofortified crops in the preparation of food in Sub‐Saharan Africa is important in fighting against problems of hunger and malnutrition. According to UNICEF (2015) and Mpofu et al. (2014), the Sub‐Saharan region suffers from inadequate intake of calories, protein, and micronutrients among children and women. This has created economic and social challenges in many communities as well as adversely affecting their productivity (Akombi et al., 2017). Poor nutrition can cause suboptimal brain development, which is associated with poor cognitive development, educational performance, and economic output in adults (Leroy et al., 2014). In the development of a child, rapid mental and physical growth occur during the first 1,000 days of life (0–23 months) which are critical in a child's life (Onis & Branca, 2016).

Undernutrition is the underlying cause of death in approximately 45% of children under five years of age (Black et al., 2013). Globally, approximately 21.3% (144.0 million) of children under 5 years of age are stunted and 6.9% (47.0 million) are wasted (FAO, 2020). African has the highest prevalence of undernutrition, with 39.4% stunted, 24.9% underweight and 10.3% wasted in children under 5 years of age (WHO, 2010). Furthermore, a 2015 Millennium development goal (MDG) report showed that 30% of all undernourished children in the world were from sub‐Saharan Africa (UN Report, 2015). TheUNICEF global nutrition 2020 report indicated a high VAD of 48% and 44% in children aged 6–59 months in 2013 was from sub‐Saharan Africa and South Asia, respectively (UNICEF, 2020). More so, the Food and Nutrition Council of Zimbabwe (2018) reported that 34% of children under 5 years of age suffer from chronic malnutrition and insufficient intakes of energy, protein, and vitamin A in many parts of Zimbabwe.

Maize (*Zea mays L*.) is an important staple food crop for many people in Africa, Asia, and South America (Yadav et al., 2015). It has an estimated total world production output of 1,060 million metric tonnes and is produced in over 160 countries (FAOSTAT, 2016). Maize per capita consumption in Zimbabwe is high and was found to be 330 g per person per day (Muzhingi et al., 2011). Maize provides more than two‐thirds of the daily energy intake in southern Africa (USAID, 2005). *Mutwiwa* or *ilambazi lokubilisa,* a fermented maize sour porridge is mainly produced at home to provide nutrition for infants in Southern Africa (Gadaga et al., 1999). Other foods such as *mahewu,* a nonalcoholic fermented beverage are also used to support human nutrition (Chawafambira et al., 2020; Matsheka et al., 2013). In the preparation of *mutwiwa*, dried WM grains are dehulled using pestle and mortar, washed, steeped in clean water, and then left to ferment for a day (Gadaga et al., 1999). The fermented maize is then dried, milled into a meal, and cooked to make a thick porridge.

The utilization of biofortified crops in the preparation of food is still limited in Southern Africa with most research focusing on crop production. Yellow maize has been perceived as a feed for animals for decades (Graham & Rosser, 2000). This perception has created a barrier to PVA‐biofortified maize consumption in Zimbabwe and has resulted in a high preference for consumption of WM (which lacks carotenoids) (Muzhingi et al., 2008). Therefore, the objectives of this study were to assess the potential of replacing WM with PVA‐biofortified maize in the preparation of traditional fermented food*, mutwiwa* enriched with milk and to evaluate the chemical composition and microbiological safety of the food product.

## 2 MATERIALS AND METHODS

### 2.1 Study area

The study was conducted in Gokwe (18.22°S 28.93°E), a communal farming area in Zimbabwe with a rich *mutwiwa* tradition. The widespread consumption of *mutwiwa* prepared using WM in the area determined the choice of this community as the study site. The area is located in Agro farming region 3 with high incidences of nutrient malnutrition especially in children, women, and the elderly.

### 2.2 Sample collection

PVA‐biofortified maize variety ZS242 was obtained from the CIMMYT Research Station, Chiredzi, Zimbabwe. The variety was chosen because it is widely available to many farmers and has been found to produce *sadza* (a local staple food) with good aroma and taste (CIMMYT/Harvest Plus, 2020, Chikulo, (2020). WM variety SC 403 popularly known as “tsoko” was obtained from households. This WM variety was chosen because most farmers grow it in the area as an early maturity maize crop. Milk was obtained from rural households in Gokwe communal area, and the milking process was done by the households and observed. Samples were collected and were analyzed for microbial safety.

### 2.3 Sample preparation and processing of PVA‐biofortified mutwiwa

**FIGURE 1:**
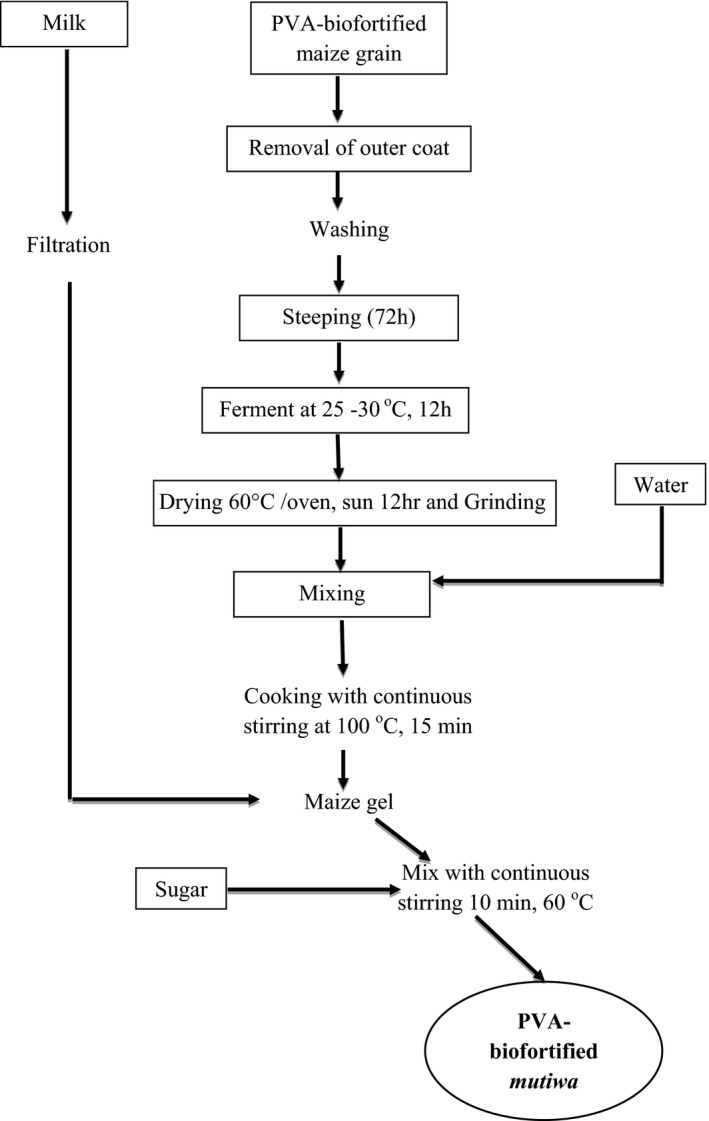
Flow chart of *mutwiwa* preparation

PVA‐biofortified *mutwiwa* was prepared according to a traditional method described by Gadaga et al. (1999) with the assistance of local households in Gokwe. Three households prepared *mutwiwa* in duplicate according to their traditional technical know‐how. In this study, PVA‐biofortified maize was used as a substitute for the indigenous WM which is commonly used to prepare *mutwiwa* by most families. The illustration of process stages used in the preparation of PVABM is shown in Figure 1. In this method, PVA‐biofortified maize grains obtained from Chiredzi were brought to the homesteads. The maize kernels were then cleaned and extraneous matter removed. The grains were dehulled, washed, steeped in water for 72 hr, fermented at 25–30°C for 12 hr in a clay pot, then dried at 60°C in an oven and for 12 hr in the sun, milled in a laboratory mill (Model RLA, 201 – 80014, Hammer mill, UK) and sieved with a size 800 µm (Model. HL 20, Gallenkamp and Co Ltd) to obtain flour. Cooking temperatures were measured by inserting a thermometer into a sample. The average amounts (*n* = 6) of ingredients were 350 g of orange maize flour, 60 g of sugar, and 485 g (500 ml) of milk. A control sample had WM. Each maize flour sample (350 g) was mixed in 500 ml of cold water in a stainless steel pot before further addition of 1.150 ml of boiling water. The mixture was cooked at 100°C for 15 min under continuous stirring until it was gelatinized. Crystalline sugar and raw milk were then added. Milk was added as an ingredient to improve the protein and mineral content of the food. The mixture was then stirred for 10 min while cooking at 60°C until a homogenous semi‐solid gel was obtained. The porridge samples were then analyzed for chemical and microbial safety.

### 2.4 Chemical analysis

Proximate analysis on moisture content using (AOAC method 925.45), crude protein using Kjeldahl (AOAC method 991.20), crude fiber using the enzymatic gravimetric method (AOAC method 985.29), crude fat using Soxhlet method (AOAC 989.05), mineral content using Inductively Coupled Plasma–Optical Emission Spectrometer (ICP‐OES) (Agilent 5,100, Agilent Technologies), and ash content using dry ashing (AOAC method 938.08) were determined according to standard methods by the Association of Official Analytical Chemists (AOAC, 2005). Total carbohydrate was determined by the difference method. The total energy content was calculated using Atwater factors (4 kcal/g for protein, 9 kcal/g for fat, and 4 kcal/g for total carbohydrate). The vitamin C (ascorbic acid) content was determined by the Dichlorophenolindophenol (DCPIP) titration test according to the AOAC method (2005). Amylose, starch, and sugar were determined using procedures by Juliano et al. (1981).

### 2.5 Amino acid analysis

Essential amino acids were analyzed and using the HCI hydrolysis process. The freeze‐dried sample was mixed with 6 N HCl in a vial. The mixture was treated with argon to remove oxygen. The closed vial was heated at 110°C for 18–24 hr and cooled. The hydrolysate was then centrifuged, filtrated, dried in Eppendorf tubes, and then reconstituted using borate buffer for derivatization. Borate buffer was pipetted into a 200 µl into 2 ml glass vial, and 10 µl of diluted sample was then added. The mixture was mixed with 6‐aminoquinolyl‐N‐hydroxysccinimidyl carbamate reagent and then vortexed to ensure proper mixing of the sample. The mixture was heated at 55°C for 10 min in an oven and loaded into an autosampler tray for analysis. The analysis was done using ultra‐performance liquid chromatography (UPLC) with a waters photodiode array detector (Waters) for high‐resolution UPLC‐UV analysis. The separation was carried out at 60°C using an Acquity UPLC BEH C18 (2.1 × 150 mm; 1.7 µm particle size) column and flowing at 0.4 ml/min. Results were obtained at a wavelength of 254 nm, and the content was expressed as g/100 g.

### 2.6 Carotenoid extraction and analysis

In determining carotenoids, extraction was done according to a method adapted from Rodriguez‐Amaya and Kimura (2004) with modifications in the extraction solvent and absorbance level. A 15 g sample was weighed on a digital balance. In extracting carotenoids, successive additions of 25 ml of acetone were added to the sample to obtain a paste. The paste was then transferred into a sintered funnel (5 μm) attached to a 250 ml flask and then filtered under vacuum. The filtration process was repeated until the sample became colorless. The extract obtained was then transferred into a 500 ml funnel containing 40 ml of petroleum ether. Ultrapure water was added to remove the acetone without precipitation and the aqueous phase was thrown away. This procedure was repeated until no residual solvent remained in the sample. The extract was then transferred into a 50 ml volumetric flask containing 15 g of anhydrous sodium sulfate. Petroleum ether was added to make up the volume to 50 ml to obtain a working carotenoid extract sample.

### 2.7 Identification and quantification of individual carotenoids

In the identification and quantification of individual carotenoids, a 2 ml sample of the carotenoid extract was dried and then diluted in 100 μl of acetone in a vortex mixer (Genie 2‐Scientific Industries). The sample was then transferred to a 2 ml amber flask for HPLC analysis. The absorbance of the sample was done at 436 nm for β–carotene. The determination of individual carotenoids was conducted using the following formula:

$$C\left(\frac{\mu g}{g}\right)=\frac{A_{X}\times C_{S}\times V\left(\text{ml}\right)}{A_{S}\times P_{g}}$$

where: *A*_x_ = Carotenoid peak area; *C*_s_ = Standard concentration; *A*_s_ = Standard area; *V* = Total extract volume and *P* = Sample weight.

### 2.8 Vitamin A analysis

The vitamin A content was calculated using the results obtained from the quantification of individual carotenoids. In the calculation of vitamin A content, a new FDA guideline (2020) on the relationship between total beta‐carotene content in a food sample and retinol activity equivalent (REA) in micrograms was used. The following formula was used: 1 µg RAE = 12 µg beta‐carotene. The vitamin A content was expressed as µg RAE.

### 2.9 Microbiological analysis

The microbial load was assessed and the type of bacteria was determined in cow's milk and prepared *mutwiwa*. Coliforms were analyzed to indicate the level of hygiene used in the preparation of the food. Salmonella was analyzed to check for any contamination of the milk and the food due to the handling of the ingredients. Yeast and molds were analyzed because there was the use of dry cereal ingredients. A 1 ml sample of raw milk was collected under aseptic conditions from the milking can and was diluted (10^–1^) using peptone physiological saline (PPS) solution (8.5 g NaCl and 1 g neutralized bacteriological peptone in 1 L demineralized water). A 1 ml subsamples were then aseptically collected and prepared into 10^–1^ dilution using peptone physiological saline (PPS) solution. An aliquot of 0.1 ml volume of dilutions was inoculated on sterile disposable Petri dishes using the pour plate method. Plate count agar (PCA), violet red bile agar (VRBA), Potato dextrose agar (PDA and deMann Rogosa Sharpe (MRS) agar were used for total viable counts, yeast and mold, coliforms, and lactic acid bacteria (LAB), respectively. A four‐stage method for *salmonella* detection was done using buffered peptone water, Rappaport‐Vassiliadis Soy Broth, and XLD (xylose lysine deoxycholate) agar. The inoculated plates with MRS agar, PDA, and VRBA were incubated at 30°C in an anaerobic jar for 72 h, 25°C for 120 h and 37°C for 24 h, respectively. PCA plates were incubated at 25°C for 48 h. LAB was confirmed by oxidase and catalase tests. Microbiological counts for each media were carried out. The estimated shelf life of the prepared *mutwiwa* sample was conducted over a 10 day period in storage at 4°C by evaluating the bacterial counts.

### 2.10 Statistical analysis

Nutritional results were analyzed using the Statistical Package for Social Science (SPSS) package version 18.0 (Coakes and Ong, John Wiley & Sons). The results were expressed as the mean ± standard deviation (*SD*). The Kruskal–Wallis nonparametric test was used to determine for any significant differences in the nutritional composition of the flours. The Mann–Whitney *U* test was used to determine the observed differences in the means of nutritional and microbiological data. Significant differences were measured at *p* < .05.

## 3 RESULTS AND DISCUSSION

A summary of the ingredients and their quantities used in preparing *mutwiwa* samples by the three households are shown in Table 1. The average yield was 950 g of PVA‐*mutwiwa* from 350 g of orange maize flour, 60 g of sugar, and 485 g (500 ml) of milk. In the control samples, WM flour was used.

**TABLE 1:** Ingredients used in the preparation of *mutwiwa* (*n* = 6) in Gokwe, Zimbabwe

Ingredients	Maximum	Minimum	Mean (*n = 6*)	*SD*
PVA‐biofortified maize (g)	400	295	350	58.3
Sugar (g)	62	51	60	10
Water (g)	2,230	1,125	1,510	178.2
Cow's milk (g)	538 (554 ml)	424 (437 ml)	485 (500 ml)	52.6
*Mutwiwa* (g)	2 623	1 546	2 010	335

Note:

WM was used as a controlled ingredient.

### 3.1 Chemical composition

The proximate composition of PVA‐biofortified maize and WM used in this study are shown in Table 2. The results showed no significant difference in percentage total moisture (*p* < .196), ash (*p* < .192), carbohydrate (*p* < .188), and sugars (*p* < .0123). However, percentage fat (*p* < .046) and crude protein (*p* < .043) contents were significantly different in the two maize genotypes (ZS242 and SC 403). Furthermore, the ratio of amylose‐to‐amylopectin in PVA‐biofortified maize was 0.191 and 0.194 in WM. This ratio was not significantly different (*p* < .05).

**TABLE 2:** Proximate composition of WM and PVA‐biofortified maize

Proximate composition (%)	WM (control)	PVA‐biofortified maize	*p*‐Value
Moisture	7.81 ± 0.2^a^	7.92 ± 0.1^a^	.196
Crude fat	5.16 ± 0.21^b^	4.46 ± 0.31^a^	.046
Crude fiber	5.11 ± 0.01^a^	4.71 ± 0.02^a^	.098
Crude protein	7.15 ± 0.11^b^	6.61 ± 0.21^a^	.043
Ash	1.28 ± 0.11^a^	1.32 ± 0.04^a^	.192
Carbohydrates	73.8 ± 0.32^a^	74.58 ± 0.04^a^	.188
Starch	89.1 ± 1.15^a^	89.6 ± 1.18^a^	.185
Sugar	7.28 ± 0.11^a^	7.47 ± 0.16^a^	.123
Amylose	14.16 ± 0.2^a^	14.03 ± 0.10^a^	.132
Amylopectin	72.9 ± 1.26^a^	73.3 ± 3.11^a^	.142

Note

Values are means ± *SD* of three determinations. Mean with different superscript letters (^a, b^) in row are significantly different (*p* < .05).

The chemical content of the *mutwiwa* samples was in the range of 4.6–5.2, 0.9–1, 0.9–1, 26.2–28.6, 62.2–66.0 g/100 g fresh weight for crude protein, crude fiber, ash, carbohydrates, and moisture, respectively (Table 3). The chemical composition was significantly different at *p* < .05 (Table 3). The results of this study have shown that PVABM is a major source of protein and carbohydrate in Gokwe. The protein content is attributed to the inclusion of milk as ingredients. Raw cow's milk contains total solids, crude protein, fat, and solids nonfat contents of 12, 3.2, 3.5, and 8.3 g/100 g, respectively. Milk composition has a direct effect on the nutritional content of the prepared PVABM. Milk is considered a near‐perfect food that contains nutritional and immunological compounds that benefit human health (Millsa et al., 2011). Milk is rich in essential proteins and vitamins that are important for human growth (Eckles et al., 2000). More so, Ladeji et al. (2000) and Nnam, (2002) have noted an improvement in protein quality in food containing milk as an ingredient and recommended its use in formulations for infants and children food. This makes the food product good and potential complementary food for infants. Pillay et al. (2013) observed a higher protein concentration in PVA‐biofortified maize than WM, whereas Oluba and Oredokun‐Lache (2018) noted a higher protein value in WM than PVA‐biofortified maize. Additionally, Sizer and Whitney, (2017) reported that maize flour can be combined with lysine and tryptophan‐rich foods so as to improve the protein quality of the diet. Milk is a good source of essential amino acids and this supports the use of milk as an ingredient in this study.

**TABLE 3:** Chemical composition of PVABM and control sample (WM)

Nutrients	PVAB‐ *mutwiwa*	WM‐*mutwiwa* (control)	*p*‐Value
Energy (Cal/100 g w.b)	154 ± 1.2^a^	134 ± 1.2^b^	<.001
Energy (kJ/100 g w.b)	647 ± 1.1^a^	563 ± 1.1^b^	<.001
Carbohydrates (g/100 g w.b)	28.6 ± 2.0^a^	26.2 ± 1.6^a^	.017
Crude Protein (g/100 g w.b)	5.2 ± 0.2^a^	4.6 ± 0.1^b^	<.001
Fat (g/100 g w.b)	2.1 ± 0.1^a^	1.3 ± 0.2^b^	.001
Crude fiber (g/100 g w.b)	1.0 ± 0.3^a^	0.9 ± 0.1^a^	.042
Ash (g/100 g w.b)	0.9 ± 0.08^a^	1.0 ± 0.1^a^	.04
Moisture (g/100 g w.b)	62.2 ± 2.1^a^	66.0 ± 3^a^	.036
Total soluble solids (g/100 g w.b)	3.6 ± 0.03^a^	3.2 ± 0.02^b^	.031
β‐carotene (µg /g w.b)	1.1 ± 0.1^a^	0.1 ± 0.01^b^	<.001
Vitamin C (mg /g w.b)	0.54 ± 0.06^a^	0.13 ± 0.01^b^	.020
Vitamin A (µg REA/100 g w.b)	9 ± 1.1	–	<.000

Note

Mean ± standard deviations are reported. Means in a row with different superscripts (^a, b^) are significantly different at *p* < .05.

The vitamin C content range was 0.13–0.54 mg/g w.b. (Table 3). The vitamin C content in the fermented food can potentially benefit children under 5 years of age but is very low for the RDA for pregnant and lactating women as well as the elderly. Furthermore, the prepared *mutwiwa* has the potential to supply over 30% and 18% of the recommended dietary allowance (RDA) for carbohydrates and proteins in children diet (1–9 years). Protein‐energy malnutrition and micronutrient deficiencies are more widespread in sub‐Saharan Africa because most of the population groups are poor and food insecure (Bain et al., 2016; Smuts et al., 2005).

The β‐carotene content was 1.1 and 0.1 µg/g w.b in PVABM and WM‐*mutwiwa,* respectively (Table 3). This study showed a relatively low β‐carotene content in the *mutwiwa* samples and this could be attributed to the effect of processing of the PVA‐biofortified maize during the preparation of PVABM. Rodriguez‐Amaya and Kimura (2004) reported that carotenoids could be damaged during food processing and storage due to enzymatic or nonenzymatic oxidation reactions. During cooking, heat treatment will cause the isomerization of transcarotenoids to their cis‐isomers and this results in the alteration of their biological activity and discolorization of the food. This might suggest the relatively low vitamin A content observed in this study.

### 3.2 Total carotenoids

The carotenoid composition of WM and PVA‐biofortified maize samples is shown in Table 4. PVA‐biofortified maize showed the presence of β‐Cryptoxanthin (1.21 ± 0.01 µg/g DW), 13‐cis‐β‐carotene (0.02 ± 0.02 µg/g DW), α‐Carotene (0.82 ± 0.01 µg/g DW), 9‐cis‐β‐carotene (0.80 ± 0.01 µg/g DW), All‐trans‐β‐carotene (0.85 ± 0.01 µg/g DW), lutein (7.56 ± 0.60 µg/g DW) and zeaxanthin (1.52 ± 0.01 µg/g DW) and WM had only lutein (1.2 ± 0.4 µg/g DW). The total carotene content of PVA‐biofortified maize was 12.78 ± 0.5µg/g DW.

**TABLE 4:** Carotenoid composition of WM and PVA‐biofortified maize (µg /g DW)

	PVA‐biofortified maize	WM (control)
β‐Cryptoxanthin	1.21 ± 0.01	–
13‐cis‐β‐carotene	0.02 ± 0.02	–
α‐Carotene	0.82 ± 0.01	–
9‐cis‐β‐carotene	0.80 ± 0.01	–
All‐trans‐β‐carotene	0.85 ± 0.01	–
Lutein	7.56 ± 0.60^a^	1.2 ± 0.4^b^
Zeaxanthin	1.52 ± 0.01	–

Note:

Data reported as means ± *SD* of three determinations. Mean with different superscript (^a, b^) letters in row are significantly different (*p* < .05).

Furthermore, La Frano et al. (2014) reported the importance of the conversion of PVA carotenoids into vitamin A in the human body. Li et al. (2010) and Muzhingi et al. (2011) noted an effective conversion of PVA carotenoids into vitamin A after the consumption of biofortified maize. Palmer et al. (2016) observed an improved serum β‐carotene level in Zambian preschool children that regularly consumed diets prepared with biofortified maize. These observations buttress the importance of using PVA‐ biofortified maize as a sustainable and effective complementary approach to deal with VAD in Zimbabwe and Southern Africa.

### 3.3 Amino acid content

The amino acid content in maize flours and prepared *mutwiwa* samples is shown in Table 5. Lysine content in PVA‐biofortified maize flour and WM was 0.38 g/100 g and 0.40 g/100 g, respectively. Isoleucine and threonine content were significantly different (*p* < .05). The prepared PVABM had a phenylalanine, lysine, and histidine content of 1.15, 0.71, and 0.56 g/100 g w.b, respectively. The Mann–Whitney *U* test results indicated that concentrations of the essential amino acids histidine, phenylalanine, and lysine were significantly different (*p* < .05) in the prepared *mutwiwa* samples. PVABM had an improved lysine, phenylalanine, and threonine concentration that was significant (*p* < .05). The results could be attributed to the addition of milk during the preparation of PVABM. Milk proteins contain nearly all essential amino acids (Mpofu et al., 2014).

**TABLE 5:** Essential amino acid composition of WM, PVA‐biofortified maize flours, and *Mutwiwa* samples

Sample	Histidine	Leucine	Lysine	Isoleucine	Methionine	Phenylalanine	Threonine	Valine
Raw milled maize flour (g/100 DW)								
WM flour	0.27 ± 0.04	0.75 ± 0.01	0.40 ± 0.03	0.58 ± 0.06	ND	0.90 ± 0.02	0.41 ± 0.04	0.42 ± 0.05
PVA‐biofortified maize flour	0.21 ± 0.03	0.68 ± 0.11	0.38 ± 0.01	0.79 ± 0.15	ND	0.80 ± 0.01	0.25 ± 0.02	0.38 ± 0.04
*p*‐Value^a^	ns	ns	ns	**.03**		ns	**.04**	ns
Food product (g/100 g w.b)								
PVABM	0.56 ± 0.05	0.65 ± 0.05	0.71 ± 0.11	0.77 ± 0.01	0.32 ± 0.01	1.15 ± 0.11	0.36 ± 0.01	0.46 ± 0.12
WM‐*mutwiwa*	0.44 ± 0.01	0.68 ± 0.03	0.58 ± 0.2	0.65 ± 0.02	0.36 ± 0.03	1.04 ± 0.02	0.38 ± 0.11	0.50 ± 0.06
*p*‐Value^b^	**.036**	.067	**.021**	.052	.076	**.04**	.053	.058

Note.

Values are means ± *SD* of three determinations; ns = not significant.

Abbreviations: DW, dry weight basis; ND, not detected; w.b, wet basis.

^a^ Student *t* test.

^b^ Mann–Whitney *U*‐test: values in bold are significantly different (*p* < .05)

### 3.4 Mineral content

The PVABM is a good source of healthy beneficial minerals needed for the proper functioning of the body (Table 6). The good mineral content could be attributed to the ingredients used in preparing the fermented food. Eckles et al. (2000) reported the presence of all 22 minerals in milk that are considered essential in human diets. The calculations on the estimated contribution of PVABM on the RDA for minerals indicated that it could potentially contribute about 212% of the Recommended Dietary Allowance (RDA) for magnesium (130 mg/100 g), 117% of the RDA for phosphorus (350 mg/100 g), 111% of the RDA for iron (7 mg/100 g ), 188% of the RDA for sodium (120 mg/100 g), and 120% of the RDA for zinc (5 mg/100 g) in children (1–9 years) when consumed as part of the diet. PVA‐*mutwiwa* was high in phosphorus and iron contents as compared to a commercial infant food formula, Cerelac® (400 mg/100 g phosphorus and 7.5 mg/100 g iron) found on the market. The iron and phosphorus content was not significantly different (*p* < .05). This meant that the PVABM can possibly be used as a source of iron and phosphorus in infants and children. Additionally, results of this study showed that PVA‐*mutwiwa* has a potential to contribute about 78% of the RDA for magnesium (350 mg/100 g), 50% of the RDA for zinc (12 mg/100 g), and 28% of the RDA for iron (27 mg/100 g) in pregnant women of all ages. A study by Pillay et al. (2013) observed a lower concentration of iron in PVA‐biofortified maize than WM but this study has shown a significant difference in the iron content between PVABM and WM (control).

**TABLE 6:** Mineral content of PVAB‐*mutwiwa* and WM‐*mutwiwa*

Mineral	Food sample		*p*‐Value
	PVAB‐*mutwiwa*	WM‐*mutwiwa*	
Iron (mg/100g w.b)	7.80 ± 0.8^a^	5.2 ± 0.5^b^	<.001
Magnesium (mg/100g w.b)	276.0 ± 1.62^a^	255.6 ± 2.31^b^	.015
Calcium (mg/100g wb)	60.5 ± 1.40^a^	50.2 ± 1.2^b^	<.001
Zinc (mg/100g wb)	6.0 ± 0.1^a^	4.2 ± 0.5^b^	.09
Potassium (mg/100g wb)	105.8 ± 3.50^a^	87.2 ± 4.23^b^	.021
Phosphorus (mg/100g wb)	410.8 ± 2.60^a^	376.5 ± 1.7^b^	<.001
Copper (mg/100g wb)	0.10 ± 0.1^a^	0.9 ± 0.2^a^	.621
Sodium (mg/100g wb)	226.4 ± 1.3^a^	208.2 ± 2.1^a^	.022

Note

Mean ± standard deviations are reported. Means in a row with different superscripts (^a,b^) are significantly different at *p* < .05.

### 3.5 Microbiological properties

The mean microbiological counts of raw cow's milk used as an ingredient were high. It was important to assay for the presence of these bacteria because the raw milk was added without any pretreatment to improve its safety. Milk is sterile when secreted in the udder but is contaminated by bacteria even before it leaves the udder (Zhang et al., 2008). Milk is potentially susceptible to contamination with pathogenic and other microorganisms which cause spoilage and illness (Eckles et al., 2000). Therefore, it became important in this study to analyze the microbial load of raw milk. The mean mesophilic bacteria counts, lactic acid bacteria, yeast , molds, and coliforms were 5.9 ± 1, 5.2 ± 1, 1, 2, and <1 Log CFU/ml, respectively (Table 7). The mesophilic bacterial count was above the regulatory level of 5.7 Log CFU/ml in milk for processing (Dairibord Zimbabwe, 2009). These results suggest the possibilities of contamination of the milk by milk handlers, their hands, the environment, filter cloth, and milking containers that might have been contaminated with microorganisms (Mpofu et al., 2014). More so, the environment temperatures at which the milking was done and storage temperatures of the milk before processing to *mutwiwa* could also have promoted the growth of bacteria. This is supported by the International Dairy Federation (1990) which reported that high temperatures experienced in tropical areas favor the growth of mesophilic bacteria. The natural antimicrobial compounds in milk, such as lactoperoxidase, lysozyme, immunoglobulins, and lactoferrins (Silanikove et al., 2006), could have tried to inhibit the growth of bacteria for a few minutes after milking (Zhang et al., 2008).

**TABLE 7:** Microbiological properties of PVABM and WM‐*mutwiwa* in storage

Sample	Mesophilic bacteria	LAB	Yeast	Molds	Coliforms	*Salmonella*
Raw milk	5.9 ± 1	5.2 ± 1	1 ± 1	2 ± 1	<1	ND
Day 0						
WM (control)	<1	<1	ND	ND	ND	ND
PVABM	ND	<1	<1	<1	ND	ND
*p*‐Value^a^		1.000				
Day 10						
WM (control)	2	6.1	6	2.2	1	ND
PVABM	3.3	7.1	5.5	2.5	1	ND
*p*‐Value^b^	.001	<.0001	<.0001	.001	.04	

Note

Mean bacterial counts Log CFU/ml.

Abbreviations: LAB, Lactic acid bacteria; ND, Not Detected.

^a^ Student *t* test.

^b^ Mann–Whitney *U* test.

The PVABM had a mean mesophilic bacteria, yeast, and mold counts of <1 Log CFU/ml, and coliforms were not detected. The drop in the microbiological counts, when compared to milk, was significantly different (*p* < .0001) as shown in Table 7. The reduction in the bacterial counts could be attributed to the heating process (>90°C) that was applied during the processing of the PVABM. Mesophilic bacteria grow at an optimum temperature range of 30–40°C (Dairibord Zimbabwe, 2009). There was a significant increase (*p* < .001) in the microbiological counts of LAB, mesophilic bacteria, yeast and molds, and coliforms during storage (4°C) at day 10 (Table 7). Gadaga et al. (1999) reported that LAB, *Pediococcus pentosaceus* were responsible for the fermentation of *mutwiwa*. Psychrotrophic bacteria might have been present and were able to grow at refrigeration conditions as indicated by microbial results.

## 4 CONCLUSION

This study showed PVA‐biofortified maize and WM a total carotene content of 12.78 and 1.52 µg/g DW, respectively. The PVA‐biofortified fermented *mutiwa* had a nutritional content of 5.2, 28.6, 2.1, 62.2, and 2.0 g/100g w.b for protein, carbohydrates fiber, moisture, and ash, respectively. Furthermore, vitamin A was 9 µg REA/100g, and consumption of PVA‐biofortified fermented *mutiwa* has the potential to improve VAD, human health, and nutrition. PVA‐biofortified fermented *mutiwa* is a good source of essential amino acids. Lysine, phenylalanine, and histidine content were significantly different (*p* < .05). More so, the iron (7.8), calcium (60.5), phosphorus (410.8), and zinc (60) mg/100 g w.b were significantly different (*p* < .05). The study noted the importance of the addition of cow's milk as it complements the nutrients especially proteins and minerals of the traditionally prepared *mutwiwa* which is normally produced with WM. Therefore, future research must evaluate the sensory acceptability and consumer perceptions of combining biofortified maize with milk as a way to produce a nutritious food with the potential of combat vitamin A and other mineral deficiencies in Sub‐Saharan Africa.

## CONFLICT OF INTEREST

The authors declare there is no conflict of interest.

## Data Availability

The data of this research is available and will be provided on request by the authors.
